# Supplementary material for: Dynalign II: common secondary structure prediction for RNA homologs with domain insertions
Source: Nucleic Acids Res. 2014 Nov 21;42(22):13939–48. doi: 10.1093/nar/gku1172 (PMC4267632; doi:10.1093/nar/gku1172)
Supplement: SUPPLEMENTARY DATA [file supp_gku1172_nar-02021-z-2014-File012.zip › manual/RNA_class/html/classes.html]

RNAstructure Classes: Alphabetical List

- Main Page
- Classes
- Files

- Class List
- Class Hierarchy
- Class Members

# Class Index

D | H | M | O | R | T

|  |  |  |  |  |
| --- | --- | --- | --- | --- |
| D | HybridRNA | Multilign\_object | R | Thermodynamics |
| Dynalign\_object | M | O | RNA | TwoRNA |
| H | Multifind\_object | Oligowalk\_object | T |  |

D | H | M | O | R | T

---

Generated on Tue Jul 30 10:52:29 2013 for RNAstructure Classes by 
 1.5.7.1
